# Tartaric Acid Used for Aiding the Solution of Disulphate of Quinine

**Published:** 1852-03

**Authors:** 


### MATERIA MEDICA AND THERAPEUTICS.

*Tartaric Acid used for aiding the solution of Disulphate of Quinine.*—A few drops of dilute sulphuric acid will, as is well known, aid to dissolve disulphate of quinine, but the taste of the mixture is then very bitter and disagreeable. Messrs. Bouchardat, Righni, and Ruspini advise tartaric acid to be used for the purpose of hastening the solution of the quinine instead of sulphuric acid; and M. Casorati has found that one grain of the tartaric acid is sufficient to saturate three of disulphate of quinine, the compound being then a sulpho tartrate of quinine, and not at all unpleasant to the taste.—*London Lancet.*

